# Methods for evaluating intersectoral action partnerships to address the social determinants of health: a scoping review

**DOI:** 10.24095/hpcdp.44.10.04

**Published:** 2024-10

**Authors:** Roshaany Asirvatham, Allison Nelson, Jonathan Northam, Kelsey Lucyk

**Affiliations:** 1 Health Equity and Policy Directorate, Strategic Policy Branch, Public Health Agency of Canada, Ottawa, Ontario, Canada; 2 Social Determinants of Health Division, Centre for Chronic Disease Prevention and Health Equity, Health Promotion and Chronic Disease Prevention Branch, Public Health Agency of Canada, Ottawa, Ontario, Canada; 3 Division of Aging, Seniors and Dementia, Centre for Health Promotion, Health Promotion and Chronic Disease Prevention Branch, Public Health Agency of Canada, Ottawa, Ontario, Canada

**Keywords:** intersectoral action, social determinants, evaluation, methods

## Abstract

**Introduction:**

Many of the social and economic factors that shape conditions for population health and health equity (e.g. income, education and employment) lie outside of the health sector. Intersectoral action (ISA) is pivotal to building diverse partnerships that address these social determinants of health. Despite the significant role of ISA, there are few comprehensive reports from the health sector on how such partnerships are evaluated. The purpose of this scoping review is to provide an overview of examples of ISA partnership evaluations, including the identification of evaluation methods, tools and indicators.

**Methods:**

A literature search of two academic databases, Embase and MEDLINE, identified seven relevant studies published between 2012 and 2022.

**Results:**

Common evaluation approaches were network analysis, community- or system-level analysis, partnership evaluation and longitudinal process evaluation. Five of the studies assessed the strength and functionality of partnerships, with reach (e.g. distance between partners) used most frequently as an indicator.

**Conclusion:**

Despite the complexity of evaluating ISA partnerships, such evaluations are crucial for assessing impacts on health outcomes and social determinants of health, goal achievement, accountability and sustainability. Different evaluation models are available to program planners and evaluators involved in ISA initiatives.

**Highlights**

- Intersectoral action is an essential approach to addressing the social determinants of health. However, literature evaluating intersectoral action is limited.
- Network analysis, system-level analysis, partnership evaluation and longitudinal process evaluation are promising approaches to evaluating intersectoral initiatives.
- Assessing the reach of intersectoral partners to each other and to the target population is important to understanding the effectiveness of intersectoral partnerships.

## Introduction

A growing body of literature reports that action on the social determinants of health, such as housing, income, employment and food security, is critical to improving population health.^1^,^2^ Intersectoral action (ISA) is a key approach to addressing complex public health challenges with the potential to impact such determinants.^3^ ISA requires that multiple sectors work together towards a common goal, which could include health equity, population health or non-health sector goals such as well-being or the environment.^3^

ISA is an important approach for health actors to promote Health in All Policies (HiAP), which sustains and influences policies supporting health and health equity.^4^ While ISA constitutes the third of the four pillars of the HiAP approach,^4^ ways of working and work methods, the value of partnerships in intersectoral collaboration warrant examination of best practices.

ISA is also recognized as a core competency for public health in Canada (“partnerships, collaboration and advocacy”).^5^ It may be particularly important for actors working in health policy settings who must collaborate to codevelop policies, mediate partners’ competing interests and advocate for health in advancing mutual goals.

Intersectoral approaches can involve partners (e.g. from government, the private sector, community organizations) from various sectors (e.g. health, education, housing, labour and employment, criminal justice) or within the same sector (e.g. government), across various departments (e.g. health, education, agriculture, finance) or jurisdictions (e.g. federal, provincial or territorial, municipal or county).

Many collaborative policy initiatives have used ISA to address complex health challenges. Governments in South Australia and Finland, among others,^4^ have implemented HiAP approaches and used ISA to integrate health considerations into the policies and programs of other sectors.^6^-^8^ For example, HiAP in South Australia involves joint governance by the central government and the health department, with the latter working closely with other government agencies to provide technical expertise and facilitate the integration of health considerations into sector-specific policies and programs.^6^ An example seen more frequently in low- and middle-income countries is the development of One Health approaches that bridge multiple sectors by focusing on the interaction between humans, animals and their shared environment and on the implications of this overlap on health.^9^

While previous reports have demonstrated the importance of ISA to public health practice^3^,^10^ and its potential positive effect on advancing health equity,^11^-^14^ there is a lack of published information on how ISA is evaluated in terms of its effectiveness in achieving intended goals, its impact on health outcomes and social determinants of health, and the tools, methods and indicators used in its execution. This literature gap may be due in part to the complexity of ISA processes and the difficulty of attributing health and related outcomes to ISA. A report on evaluation methods for ISA implementation discussed a breadth of strategies for use by policy makers (e.g. increasing political will, sustaining effective processes, designing and implementing interventions), noting that each be subjected to an individual evaluation in its own right.^15^

The purpose of this scoping review is to evaluate the effectiveness of ISA, particularly focusing on the effectiveness of partnerships within ISA, hereafter referred to as “ISA partnerships,” by providing an overview of evidence-based methods and key findings from recent peer-reviewed public health literature. Evaluating the effectiveness of ISA partnerships may involve determining how partners collaborate to achieve their goals and measuring impact, communication and overall success of intersectoral efforts in addressing complex issues.

The objectives of this review are to: (1)consolidate domestic and international examples of ISA partnership evaluations; (2) identify methods, tools and indicators used in ISA partnership evaluations; and (3) understand the barriers and facilitators to evaluating ISA partnerships.

## Methods

We searched health-related literature to identify studies that explicitly evaluated ISA partnerships for their effectiveness, including their impacts on health outcomes and social determinants of health, following the 2020 guidelines developed for scoping reviews by JBI.^16^ We also followed the Preferred Reporting Items for Systematic reviews and Meta-analyses extension for Scoping Reviews (PRISMA-ScR) checklist.^17^

***Search strategy***

We searched two electronic databases, Embase and MEDLINE, in February 2022, for relevant academic literature published between 2012 and 2022. Our search combined terms from two themes: “intersectoral action” and “program evaluation,” on the recommendation of a health sciences librarian who developed our search strategy. This search was applied to both title and abstract keywords and subject headings, and was limited to peer-reviewed articles published in English within the last 10 years to ensure recency of retrieved reviews.^11^-^13^ The search strategy for MEDLINE is shown in Table 1; the search strategy in Embase used the same search terms and limits.

**Table 1:** MEDLINE database search strategy

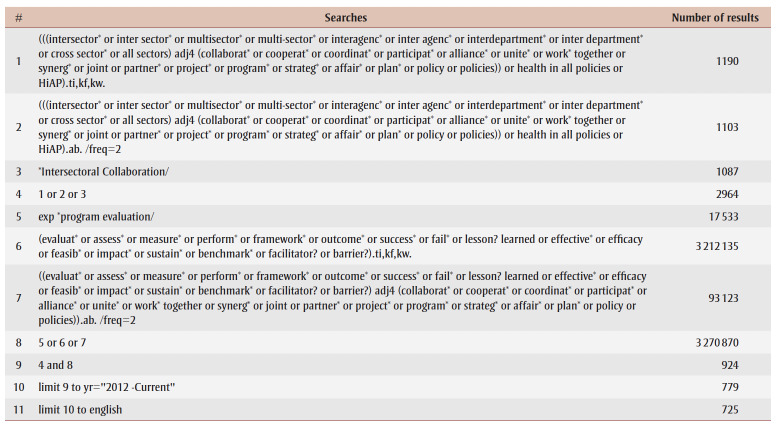

***Study selection***

Articles were eligible for review if they:

(1) reported on an ISA intervention or ISA-related activity that addressed at least one social determinant of health and involved the collaboration of two or more sectors, as determined by the coauthors during screening of the titles and abstracts of retrieved records; and

(2) explicitly mentioned the evaluation of ISA partnerships by assessing their effectiveness, successes and challenges or through other means.

Therefore, results of interest included ISA partnerships that involve two or more sectors, including but not limited to health, education and social services.

Outcomes of interest included the evaluation of ISA partnerships, at community, regional or national levels, with a focus on effectiveness, functionality and identified impacts on health outcomes and social determinants of health.

In order to examine a broad range of relevant empirical studies, we only excluded those studies that did not evaluate an ISA partnership or ISA intervention or that only examined outcomes (versus the ISA process). We also excluded clinical trials, conference abstracts, commentaries and literature reviews. 

Three authors (RA, AN, KL) independently conducted abstract screening on duplicate samples of articles (n=50) to ensure consensus and agreement on eligibility criteria. Once the eligibility criteria were established, the principal author (RA) screened the remaining abstracts (n=899).

Next, two authors (RA, JN) conducted the full-text review of 28 articles using a data extraction form developed by RA. Descriptive data extracted from the articles included the country of study; the target population; the ISA program (including relevant activities/strategies and outputs); and outcomes. Data on the evaluation of ISA initiatives included the evaluation objective; the type of evaluation (e.g. process, impact); methods and tools used for evaluation; indicators of ISA (e.g. reach, network membership or interaction, systems change, etc.); conceptualization of effectiveness; evidence for results achieved because of ISA; and barriers and facilitators to ISA evaluation. We used qualitative content analysis, following the methods of Krippendorff,^18^ to identify commonalities and themes to do with the types of evaluations, methods and tools used to assess intersectoral collaboration. Data management and coding of themes was conducted using Microsoft 365 Excel (Microsoft Corp., Redmond, WA, US).

## Results

***Search strategy results***

**Figure 1:**
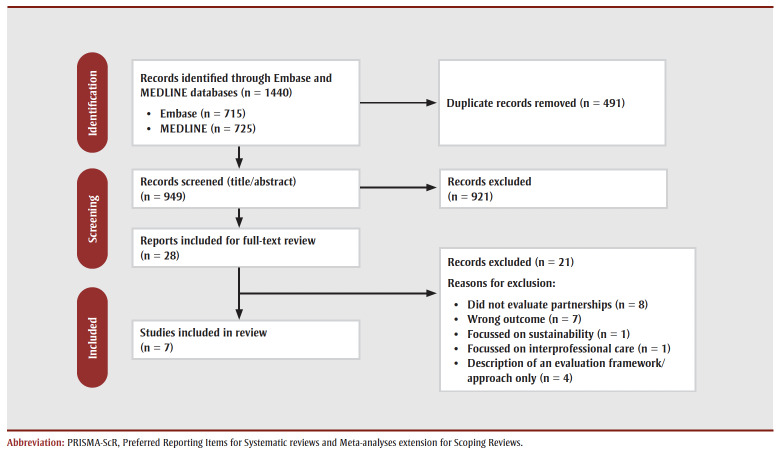
PRISMA-ScR flow diagram17 of search strategy methods

We identified 1440 articles based on the search strategy (see Figure 1). After the removal of duplicates, 949 articles underwent title and abstract screening and 28 articles were identified for full-text review. After full-text review, we included seven articles in this scoping review. These were published between 2013 and 2018, and included evaluations from Australia, Canada and the United States.

***Models and indicators***

Evaluated intersectoral interventions were in the areas of healthy eating, physical activity, child health, injury prevention, geriatric care, health promotion and chronic disease prevention, and more broadly, health equity. The range of sectors or partners involved in the intervention also differed depending on the focus of the program or on program activities. For example, a multisector collaborative in Minnesota, US that served as a bridging hub for coordinating the delivery of health promotion programs was evaluated with partners from the health care system, community-based organizations and public health.^19^ Another example included a cooperative with partnerships across primary care, mental health services, community support services and other local community agencies to coordinate cross-sectoral services to older adults.^20^

An overview of models and indicators used in the studies included in this scoping review is provided in Table 2.

**Table 2:** Indicators and models used in the evaluation studies (n = 7)

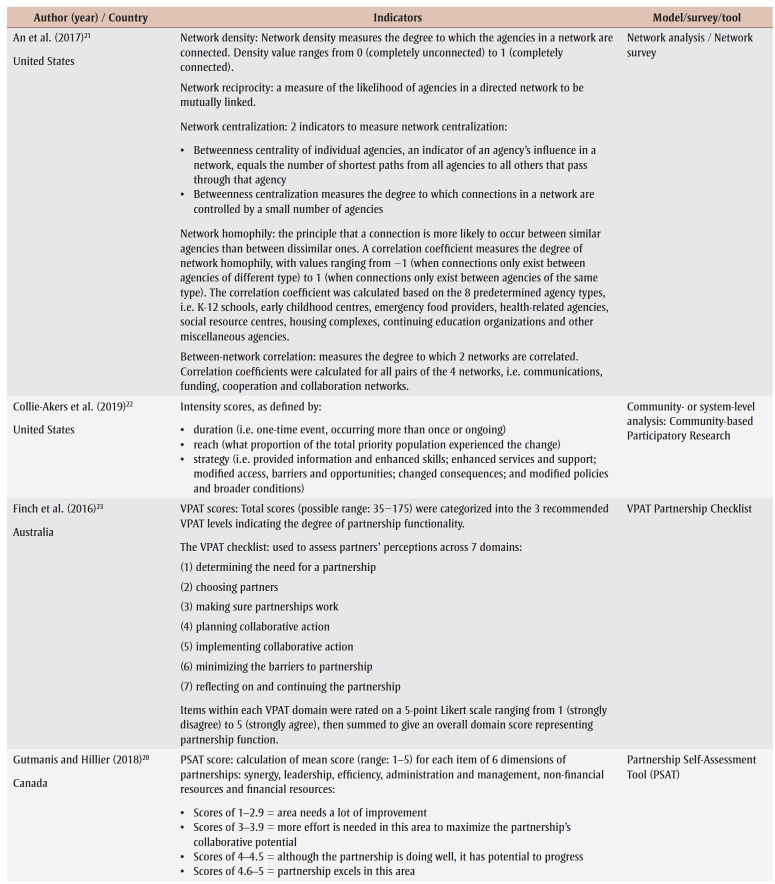 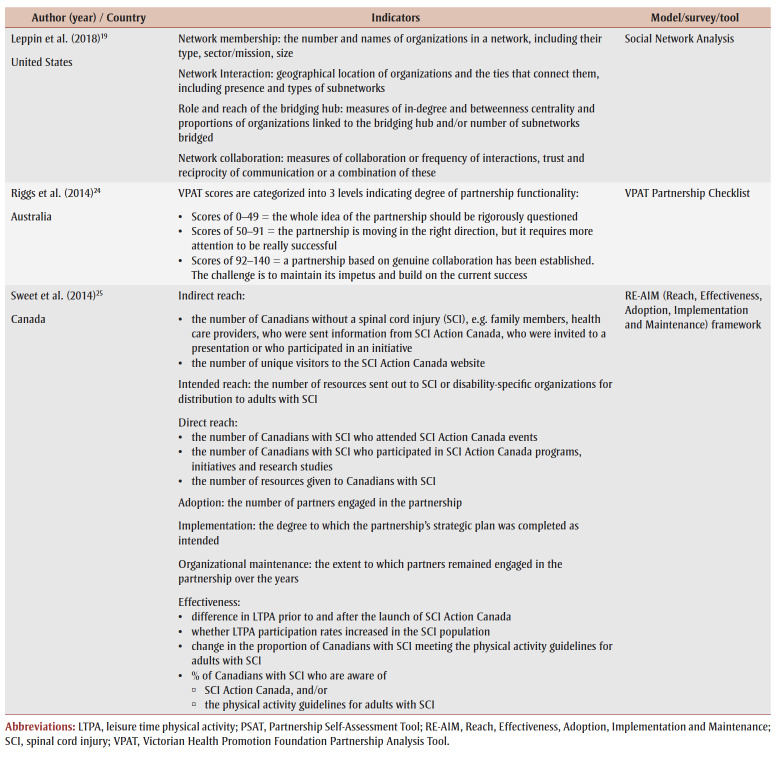

***Evaluation types and focus***

The types of evaluation differed depending on the goals and outcomes measured. Two studies used a network analysis approach to map and assess the relationships and connectedness between individuals or organizations in a network.^19^,^21^ Network analysis identifies patterns and dynamics of individuals or organizations within a network, yielding insights into the overall network structure, influential entities and flow of information, resources or influence.^21^ Collie-Akers et al.^22^ used a community- or systems-based evaluation approach to measure the progress of a collaborative health promotion coalition in Kansas, US that had the goal of reducing health inequity in a low-income neighbourhood. Two studies evaluated partnerships by assessing their strength,^20^,^23^ while one assessed partnership functioning over time via a longitudinal process.^24^ One study used the Reach, Effectiveness, Adoption, Implementation and Maintenance (RE-AIM) framework to assess the impact of intersectoral partnerships within SCI Action Canada, a large health initiative that promotes physical activity among people with spinal cord injuries.^25^ This study combined activity-specific with additional approaches, so that in some cases partnership activities were evaluated based on the RE-AIM dimension targeted, and in others, data were gathered across activities to assess other RE-AIM dimensions (e.g. reach).

Overall, the evaluation approaches that most of the studies used did not focus primarily on measuring the direct impact of the intersectoral initiative on health outcomes. Five of the studies assessed the strength and functionality of partnerships,^20^,^22^-^25^ such as identifying areas for improvement in planning or collaborative action implementation; three assessed intervention partnerships over time;^20^,^23^,^24^ and the remainder focused on mapping partnership structure and links to demonstrate the strength of relationships between partners.^19^,^21^

***Evaluation methods and analyses***

Five studies^19^-^22^,^25^ used quantitative methods while the remaining two used mixed methods. Five studies used survey designs, including a network survey,^21^ the Victorian Health Promotion Foundation Partnership Analysis Tool (VPAT) Partnership Checklist,^23^,^24^ the Partnership Self-Assessment Tool (PSAT)^20^ and a level of collaboration survey.^19^ Where quantitative data were collected and analyzed, studies used *t* tests and variance estimates to interpret findings. Three studies^20^,^23^,^24^ used ranking or scores for different survey items. One of these studies used the PSAT to calculate a mean score for items related to six dimensions of partnerships, that is, synergy, leadership, efficiency, administration and management, non-financial resources and financial resources.^20^ The mean score indicated how well collaborative processes were working, with high scores indicating strength and low scores indicating a need for improvement. Similarly, two studies^23^,^24^ that used the VPAT to assess the effectiveness of partnerships calculated aggregate scores across key factors for partnership development and success including the need for partnerships, choosing appropriate partners and making sure these partnerships are effective, among others.

Most studies that used surveys included a qualitative component to add context and understanding to quantitative findings. For example, Riggs et al.^24^ conducted a longitudinal mixed methods partnership evaluation over 2.5 years using three components: organizational ethnography, partnership survey and semistructured interviews. The authors observed partnership and staff meetings and program classes, and interviewed partners on their understanding of the partnership’s purpose, role, strengths and challenges.^24^

Four studies^19^,^21^,^22^,^24^ included visual displays, such as network maps and graphs, to show collaborative progress or partnerships. Studies that conducted network analyses showed the strength of ties between partners using multidimensional scaling and algorithms.^19^,^21^ In their network analysis, An et al.^21^ also assessed partnerships using network characteristics, namely density, reciprocity, centralization and homophily.

***Indicators and use of scores***

All of the studies included in this scoping review used indicators for evaluation and/or scores to assess partnership collaboration. The most common indicator, “reach,” used by four studies, evaluated the level of engagement or participation of the target population or partners involved in the ISA initiative. In the studies that used a network analysis approach,^19^,^21^ “reach” referred to the extent of engagement or connections between partners or organizations within a network, with the “betweenness centrality indicator” signifying the length of the path between the partners or organization and all others that pass through the network, with a shorter path indicating more direct influence. “Network density,” which refers to the extent to which partners or organizations within a network are connected to one another through relationships or collaborations, was also used as a measure to assess network-level reach.^21^

Two studies examined the reach of the target population through intersectoral initiative activities.^22^,^25^ Collie-Akers et al.^22^ examined the impact of a collaborative health promotion coalition on low-income residents by measuring the percentage of the study population that experienced a community or system change. For example, the authors reported that at least 21% of the population was exposed to community change as a result of the conversion of an underutilized park into a soccer field. Sweet et al.^25^ evaluated SCI Action Canada using the RE-AIM framework, with reach measured using three different indicators: indirect reach, intended reach and direct reach.

Two studies used VPAT scores to assess partner perceptions across seven domains, that is, determining the need for a partnership, choosing partners, making sure partnerships work, planning collaborative action, implementing collaborative action, minimizing the barriers to partnership, and reflecting on and continuing the partnership.^23^,^24^ The scores were then categorized into three levels, indicating the degree of partnership functionality from weak to strong. Similarly, Gutmanis and Hillier^20^ used PSAT scores to identify the strengths, weaknesses and synergies of partnerships across the areas of leadership, efficiency, administration and management and sufficiency of resources. Similar to VPAT, PSAT scores are categorized into levels indicating how well the partnership excels in each area.

***Barriers and facilitators to evaluating intersectoral programs***

Barriers and facilitators across the studies affect the process of assessing intersectoral initiatives. Given that most evaluation tools used surveys with self-reported responses, results were subject to measurement error, recall bias, nonresponse bias, missing/inaccurate data and other limitations. Several studies noted how difficult it was to obtain high response rates to surveys, either due to an inability to reach participants or because participants were not interested in responding or lacked the time.^19^-^21^

Another challenge reported by the studies included in this scoping review^20^,^21^,^23^ and elsewhere^26^ was the lack of consistent and long-term partnerships, complicated by changing representatives and leadership turnover. In addition, survey tools were limited to addressing changes in partnerships; for instance, Riggs et al.^24^ found that the views of respondents had changed between the partner survey when the data were first collected and a subsequent qualitative interview. Other complex factors in ISA initiatives are those that influence relationship and partnership building, such as perceived authority and empowerment in the decision-making process.^20^

Despite the drawbacks to evaluating intersectoral initiatives, common facilitators promoting the use of tools included their usability, cost-effectiveness and minimal time commitment for participants.^20^ Visualizations facilitated reflection and understanding, and allowed for adjusting efforts if needed.^22^ Some studies also reported positive discussions on evaluation results to do with communication, roles and responsibilities, shared interests and trust.^23^,^24^ The use of qualitative methods, such as ethnography, in some cases helped to build rapport and trust with participants by ensuring that evaluation findings were relevant and useful to all partners.^24^

## Discussion

Evaluating the effectiveness of ISA partnerships provides critical insights for enhancing collaboration and improving the overall effectiveness of ISA in addressing complex public health challenges, as a necessary element of HiAP implementation. We identified four examples of evaluations that assess the effectiveness of ISA partnerships from Australia, Canada and the United States. The most common evaluation approach was network analysis. Other evaluation approaches were community- or system-level analyses, partnership evaluations and longitudinal process evaluations. Through this scoping review, we also identified various tools used in evaluations including network surveys, VPAT, PSAT and level of collaboration surveys.

Our findings show that evaluation is important for assessing partnership effectiveness, accountability and transparency while promoting learning within collaborative partnerships. Specifically, evaluation helps to assess the effectiveness of certain partnerships in achieving project outcomes and goals, which can increase health actors’ understanding of partners’ policy priorities, strengthening codevelopment processes.^4^ Evaluations can also improve knowledge of partners’ areas of expertise, further building trust and improving communication between partners during collaborations and negotiations.^4^

This scoping review revealed that qualitative methods such as semistructured interviews can help promote partner engagement by including the partners’ views on what they considered effective in achieving their goals.^24^ This collaborative approach can encourage a sense of ownership and responsibility for the evaluation findings and recommendations. In addition, evaluation tools can hold partners accountable and promote transparency by clearly describing partnership roles and conditions for success. This finding is consistent with reports on HiAP development, implementation and evaluation in which key factors such as accountability, transparency and sustainability are embedded.^27^,^28^

The aim of this scoping review was to help fill the knowledge gap on how program planners and evaluators can assess the dynamics of intersectoral partnership collaboration as a key component of HiAP, the factors that contribute to effective collaboration and the barriers and challenges. The seven studies included in this scoping review were conducted in countries where ISA is increasingly recognized as a key strategy for population health and health equity, namely Australia,^23^,^24^,^29^ Canada^20^,^25^,^30^ and the United States.^19^,^21^,^22^,^31^ While the sample of articles analyzed is not large enough to determine trends or draw conclusions, the focus of work from these settings aligns with recent policy developments that cross sectors and involve the coordinated efforts of departments and ministries to jointly act on health or other well-being outcomes (e.g. South Australia’s 2007 HiAP program,^7^ Canada’s 2021 Quality of Life Strategy^32^). As such, the results not only highlight the proliferation of intersectoral initiatives, but also demonstrate the importance of implementing ISA effectively to achieve desired goals.

Despite this promise and the development of new intersectoral initiatives internationally,^6^ there remains a gap in the evaluation of intersectoral efforts in the academic literature. The reasons for this gap may include the difficulty of attributing successful outcomes of ISA initiatives to their partnerships as well as the complex nature of issues being addressed that require long-term observation. The literature in this scoping review described various models that can navigate the complexity of ISA partnerships and demonstrate the value of these initiatives even if direct outcomes cannot be attributed to specific partnerships. The model most commonly used by the studies in this scoping review was network analysis, possibly because of its strength in evaluating relationships between network members and in examining how relationships impact program outcomes.^19^,^21^ The ability of network analysis to produce a comprehensive map of an intersectoral network, including its key members, connections and communication patterns, can be useful for evaluating intersectoral initiatives.^19^ The Esmaili et al. review also found network analysis, including social network analysis, to be one of the most commonly used models of intersectoral collaboration.^33^

Some of the tools identified in this scoping review may help to overcome some of the challenges health actors face when seeking to collaborate with other sectors. “Health imperialism,” or the overemphasis of health in intersectoral policy goals,^34^ can create conflicts to do with resources, values and divergent interests in policy codevelopment, and is a recognized barrier to developing ISA partnerships.^4^ Some evaluation tools, like the PSAT, offer insights into areas where conflicts within a partnership may arise, such as in communication and information sharing, roles and responsibilities, the decision-making processes, and the adaptability and flexibility of partners.^20^ Although the use of this tool can shed light on potential conflicts, strategies to negotiate these differences are still needed. To support new ways of implementing HiAP, for example, the World Health Organization suggests being prepared to negotiate, being flexible and adaptable, and creating platforms for dialogue and problem solving related to policy, among other strategies.^4^

It is also important to note the benefits and drawbacks to the types of data utilized in evaluations. Network analysis, for instance, does not assess qualitative aspects that are vital to collaborations (e.g. trust, shared values, power dynamics) but focuses on the structure of partnerships. Triangulating quantitative data with qualitative data can therefore offer a more comprehensive understanding of intersectoral partnerships. For example, Riggs et al.^24^ added a qualitative dimension to their evaluation by using an organizational ethnography approach as well as semistructured interviews; this led to a more in-depth analysis of their survey results. Across the studies, partners’ views on key components of effective partnerships, including trust, respect and open communication, were found to improve program delivery, enhance long-term relationships and facilitate partners’ consensus on decisions.^23^,^24^ These findings also align with existing literature on facilitators to intersectoral collaboratives, where trust, respect and open communication between partners were highlighted.^35^-^37^ It is therefore important to establish key characteristics of an effective partnership at the outset of plans to evaluate partnerships.

***Strengths and limitations***

The primary limitation of this study is the focus of the search strategy on ISA, a term and concept stemming from the health field. The use of the two particular databases may have resulted in health literature and health sector findings being overrepresented. It is possible that studies from non-health sectors unfamiliar with health sector terminology used alternative terms to describe ISA initiatives that were not captured in the search. In addition, our search of peer-reviewed literature may have missed relevant work from grey literature sources. There may be relevant findings, for example, from community settings. As no pertinent literature reviews in this topic area were identified prior to undertaking this work, we did not systematically sample from the reference lists of existing reviews.

Finally, it is important to acknowledge that ISA is insufficient as a measure for assessing the effectiveness of HiAP. While ISA captures the collaborative ways of working that are critical to HiAP implementation, an evaluation of HiAP would require assessing the other pillars: governance and accountabilities, leadership at all levels and resources, financing and capabilities.^4^ However, ISA does represent an important step towards the codevelopment of healthy public policies, and we hope that this scoping review encourages groups who have evaluated ISA partnerships to publish their results and add to the evidence base.

Despite these limitations, this study has shown there to be a significant gap between the number of studies reporting on ISA initiatives and the number of studies that evaluated these efforts (i.e. seven full-text articles). Our findings are anticipated to be of use mainly for those working in the health sector.

## Conclusion

More evaluations of ISA initiatives are needed in order to better understand the effectiveness of these initiatives so that they may contribute to the implementation of HiAP approaches for health and health equity.

We encourage program managers and practitioners who have completed evaluations to share their findings broadly. Each ISA initiative has unique challenges, and it is beneficial to learn how various projects were able to respond to these in different settings. We also suggest conducting more work on the evaluation of ISA initiatives that focus on:

- How to address changes in partnerships when evaluating their impact and how to withstand these changes throughout implementation;
- How to integrate equity, diversity and inclusion into evaluations; and
- How to take into account the impact of structural and social determinants of health on ISA partnerships (e.g. power dynamics, examining the representation and participation of diverse interested parties, resource distribution, decision-making structures, etc.).

In this article we identified methods, tools and indicators that program managers, researchers and program evaluators can use when conducting evaluations on intersectoral partnerships. Evaluations in this area have the potential to identify disparities and promote evidence-based decision-making. By identifying areas for improvement within ISA partnerships, evaluations play an important role in advancing equitable health outcomes for diverse populations.

## Acknowledgements

The authors would like to acknowledge the Health Canada Library for its assistance in the literature search. The authors would also like to thank Beth Jackson for their guidance during the data analysis stage and Lucina Rakotovao for their review and feedback. We also thank the reviewers for their recommendations on how to strengthen this scoping review and our presentation of findings.

## Conflicts of interest

The authors have no conflicts of interest to declare.

## Authors’ contributions and statement

RA: Conceptualization, methodology, writing – original draft, writing – review & editing.

AN: Conceptualization, methodology, supervision, writing – review & editing.

JN: Methodology.

KL: Methodology, supervision, writing – original draft, writing – review & editing.

All authors have reviewed and approved the final manuscript.

The content and views expressed in this article are those of the authors and do not necessarily reflect those of the Government of Canada.
